# Metabolic network structure and function in bacteria goes beyond conserved enzyme components

**DOI:** 10.15698/mic2016.06.509

**Published:** 2016-04-14

**Authors:** Jannell V. Bazurto, Diana M. Downs

**Affiliations:** 1 Department of Microbiology, University of Georgia, Athens, GA 30602, USA.

**Keywords:** metabolic network, metabolic integration, plasticity, thiamine synthesis, phosphoribosylamine (PRA), phosphoribosylpyrophosphate amidotransferase (PurF)

## Abstract

For decades, experimental work has laid the foundation for our understanding of the linear and branched pathways that are integrated to form the metabolic networks on which life is built. Genetic and biochemical approaches applied in model organisms generate empirical data that correlate genes, gene products and their biological activities. In the post-genomic era, these results have served as the basis for the genome annotation that is routinely used to infer the metabolic capabilities of an organism and mathematically model the presumed metabolic network structure. At large, genome annotation and metabolic network reconstructions have demystified genomic content of non-culturable microorganisms and allowed researchers to explore the breadth of metabolisms *in silico*. Mis-annotation aside, it is unclear whether *in silico* reconstructions of metabolic structure from component parts accurately captures the higher levels of network organization and flux distribution. For this approach to provide accurate predictions, one must assume that the conservation of metabolic components leads to conservation of metabolic network architecture and function. This assumption has not been rigorously tested. Here we describe the implications of a recent study (MBio 5;7(1): e01840-15), which demonstrated that conservation of metabolic components was not sufficient to predict network structure and function.

Biosynthesis of the coenzyme thiamine pyrophosphate (TPP) in *Salmonella enterica* has served as a model system to probe metabolic integration and define network architecture *in vivo*. When this system is constrained, it is highly sensitive to slight changes in the metabolic network, and subtle changes in carbon flux can be monitored using growth analyses. A large part of our work has focused on defining metabolic redundancy and recruited pathways that can produce the first shared purine/thiamine synthesis intermediate, phosphoribosylamine (PRA). PRA is synthesized by phosphoribosylpyrophosphate amidotransferase (PurF) (Figure 1) and in a *purF* mutant, six alternative mechanisms for synthesizing PRA sufficient for thiamine synthesis have been defined experimentally. As PurF is feedback inhibited by purines, bypassing PurF with alternative or recruited pathways would decouple thiamine synthesis from regulation by purines. Such a scenario is significant when the natural habitat of *S. enterica* is expected to have substantial levels of purines due to lysed cells and degraded DNA. However, the extent to which these alternative pathways contribute to thiamine synthesis in wild type *S. enterica* has remained elusive. The following fundamental questions arose during the course of the aforementioned studies: i) are metabolic network structures dissected by mutational analysis biologically relevant? ii) if metabolisms contain identical components, is the network structure and function predetermined? In other words, does the conservation of metabolic components demand the conservation of the network? To begin to address these questions we took a comparative approach and queried the metabolic network surrounding PRA synthesis in *Escherichia coli*, a close relative of *S. enterica* with conserved metabolic pathways and regulatory paradigms. Importantly, all metabolic components relevant for PRA synthesis in *S. enterica* are present and are > 95 % identical in *E. coli*.

**Figure 1:**
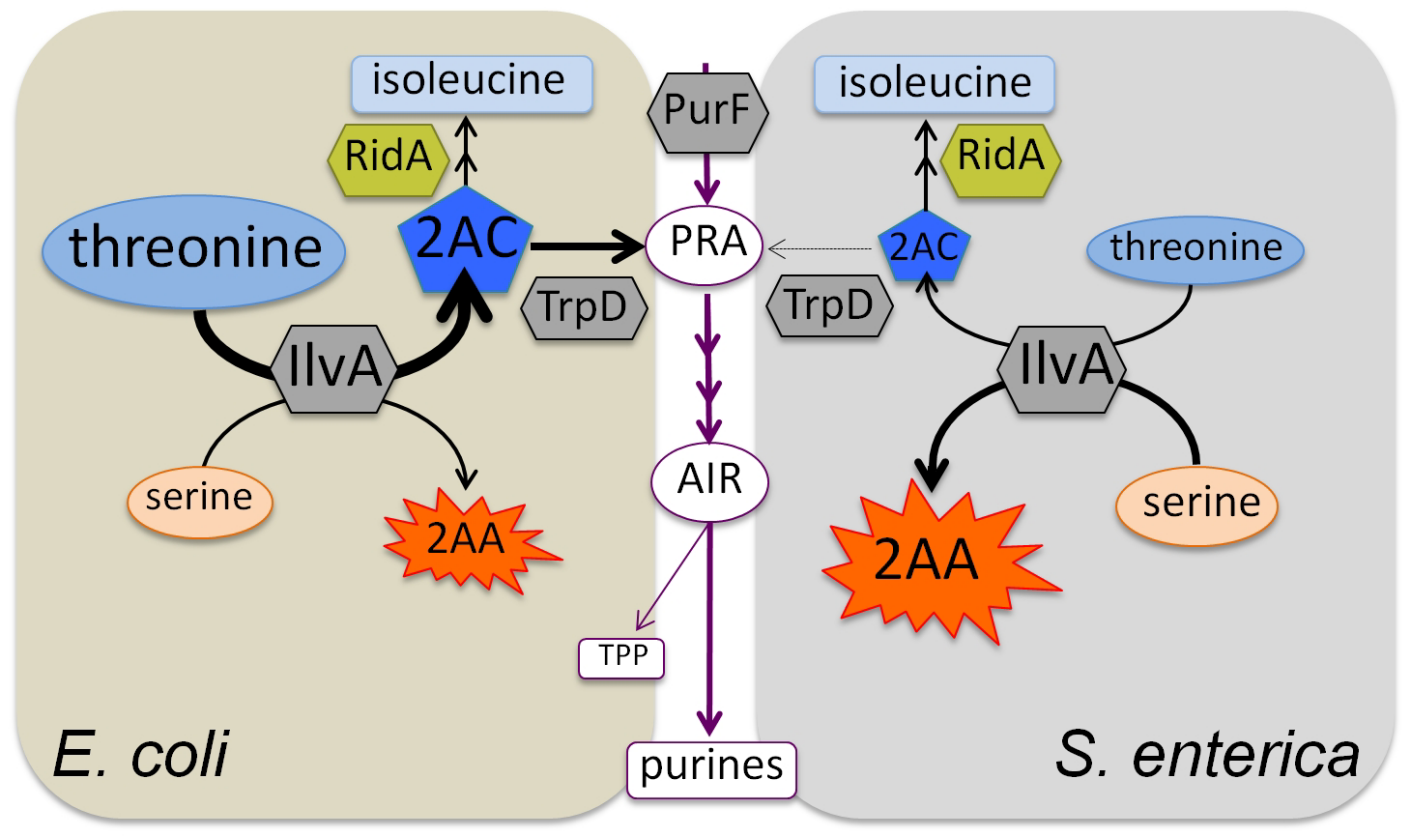
FIGURE 1: *S. enterica* and *E. coli* have distinct metabolic network structures surrounding thiamine pyrophosphate (TPP) biosynthesis. The metabolic network structures leading to IlvA/TrpD-dependent TPP synthesis in *S. enterica* and *E. coli* are shown. Notably, although *E. coli* and *S. enterica* have the same enzymes, TPP biosynthesis from threonine is only detectable in *E. coli*. A working model suggests that relative levels of threonine and serine, the substrates for IlvA, influence the structure and function of the metabolic network. The purine/TPP pathway is shown in maroon with the first enzyme, PurF, and relevant branch metabolites, PRA and AIR, indicated. The thickness of the arrows is meant to represent carbon flux. The two intermediates in the IlvA reaction mechanism, 2-AC and 2-AA are shown. The diversion of 2-AC to PRA formation is allowed by the concentration of threonine available to IlvA in *E. coli*. In our model, we hypothesize 2-AA from serine is more prevalent than 2-AC in *S. enterica*, due to the lower level of threonine available for IlvA.

Our initial results from a study to address the conservation of metabolic structure between *S. enterica* and *E. coli* were recently reported in mBio (MBio 5;7(1): e01840-15) and are summarized and expanded here. As expected, deletion of *purF* in either *E. coli* or *S. enterica* results in a requirement for exogenous purines for growth. However, the *purF* mutants of *E. coli* and *S. enterica* differ in their ability to synthesize thiamine. Both organisms maintain thiamine synthesis due to non-enzymatic formation of PRA from ribose-5-phosphate (oxidative pentose phosphate pathway) and ammonia. When the non-enzymatic mechanism is eliminated, *E. coli* retains PRA synthesis, while growth of *S. enterica* becomes dependent on exogenous thiamine. Further experimentation showed that *E. coli* utilizes an unusual pathway stitched together from isoleucine and tryptophan biosynthetic enzymes. We learned that in *E. coli*, threonine is converted to 2-aminocrotonate (2-AC) by serine/threonine dehydratase (IlvA, isoleucine biosynthesis), which is then condensed with phosphoribosylpyrophosphate by anthranilate synthase component II (TrpD, tryptophan biosynthesis) to form PRA (Figure 1). Importantly, results of labeling studies ([15N]-threonine) showed that, in *E. coli*, IvA/TrpD-dependent synthesis contributes significantly (~ 35 %) to the thiamine pool in a wild type strain. Therefore, the pathway described for canonical thiamine synthesis contributes little more than half of the cell’s total thiamine under some growth conditions. This finding was unexpected since it was not apparent from the knowledge of metabolic components present. This work demonstrated that *S. enterica* and *E. coli* do not use the same set of pathways to synthesize thiamine, and highlighted the fact that there is something fundamentally different about the network determinants of the two organisms. We can then conclude that even in closely related organisms, conserved metabolic enzymes do not guarantee conserved structure and function of the metabolic network.

The IvA/TrpD-dependent pathway for PRA synthesis was first identified in *S. enterica* as a pathway activated by a suppressor mutation (*ridA*) in a *purF* strain. *ridA* encodes the reactive intermediate deaminase, RidA, a universally conserved metabolic stress protein. RidA protects critical PLP enzymes (e.g., serine hydroxymethyltransferase) by quenching the reactive enamine 2-aminoacrylate (2-AA) generated by other PLP enzymes such as IlvA (when serine is the substrate). The threonine-derived product of IlvA, 2-AC, that is utilized by the IvA/TrpD-dependent PRA biosynthetic pathway is also a substrate of RidA (Figure 1). The fact that this pathway is active in wild type *E. coli*, but must be activated by a *ridA* lesion in *S. enterica*, suggests that 2-AC does not accumulate in *S. enterica* because of the presence of RidA. Then the question is, why does 2-AC accumulate in *E. coli*, which also has a functional RidA? In other experiments we have shown that diverse phenotypes caused by 2-AA damage in a *S. enterica ridA* strain are not observed in an *E. coli ridA* strain (unpublished data). Together our observations suggest that wild type *E. coli* makes more 2-AC and less 2-AA than *S. enterica*, implicating IlvA activity as the key difference. We hypothesize that a metabolite balance (e.g., higher threonine/serine ratio in *E. coli*) is responsible for the multiple phenotypic differences (i.e., network configurations) between the two organisms. Calculations of kinetic parameters suggest this is a feasible explanation. Advantageously, we have the genetic tools to manipulate both *E. coli* and *S. enterica*. Future efforts will attempt to “switch networks”, that is, convert the *E. coli* network into the *S. enterica* network (with respect to PRA synthesis) and vice versa.

A natural extension of metabolic studies is the implementation of mathematical models to capture the inherent complexity of biological systems. One of the current challenges in this effort is to move beyond a focus on high-flux metabolic pathways and incorporate the subtle intricacies of a metabolic network into a series of mathematical models with increasing validity. These intricacies are often responsible for the plasticity that is characteristic of metabolic networks, and they are critical for the responsiveness a network has to perturbations by external and internal signals. To accurately predict the phenotypes we observe, a productive modeling approach has to be rigorously applied to a small-scale network. Toward this end, in collaboration with Eberhard Voit and colleagues (Georgia Tech), we are developing the metabolic node that includes the purine, histidine and TPP biosynthetic pathways into a single system that can be experimentally and theoretically queried. This node is composed of low and high flux pathways and extensive points of metabolic crosstalk. We envision developing the purine/histidine/TPP node of metabolism as a model system to integrate mathematical modeling, quantitative metabolite measurements and experimental approaches that will uncover fundamental properties of the metabolic network. The integration of experimental and theoretical approaches planned may provide dynamic models to address critical questions at the interface of the physical and biological sciences. We expect to pinpoint network behaviors with a level of detail that is not yet possible for a metabolic system at a whole-cell level.

The IlvA/TrpD-dependent PRA pathway that contributes to TPP biosynthesis in wild type *E. coli* was originally characterized in mutant analysis of *S. enterica*, a strategy that is sometimes dismissed as lacking physiological relevance. The biochemical and genetic characterization of this pathway identified hallmark behaviors that were easily detected in *E. coli* and led to the quick identification of the relevant pathway. The resultant metabolic insights, that transcend the organism of discovery, underscores that the role of genetic analysis of mutants is to better understand wild type cell behavior and uncover metabolic paradigms. The most dramatic point made by this work is the recognition that metabolisms that contain the same components do not necessarily configure them the same way. This study emphasized the value in going beyond the cataloging of component parts as we strive to understand network structure, and the critical role that experimental approaches still have in deciphering cellular metabolism.

